# For Male *Caenorhabditis elegans*, Sperm Activation Is a “Just-in-Time” Event

**DOI:** 10.1371/journal.pgen.1002392

**Published:** 2011-11-17

**Authors:** Diane C. Shakes

**Affiliations:** Department of Biology, College of William and Mary, Williamsburg, Virginia, United Stated of America; University of Medicine and Dentistry of New Jersey, United States of America

## Sex-Specific Sperm Activation?

**Figure 1:**
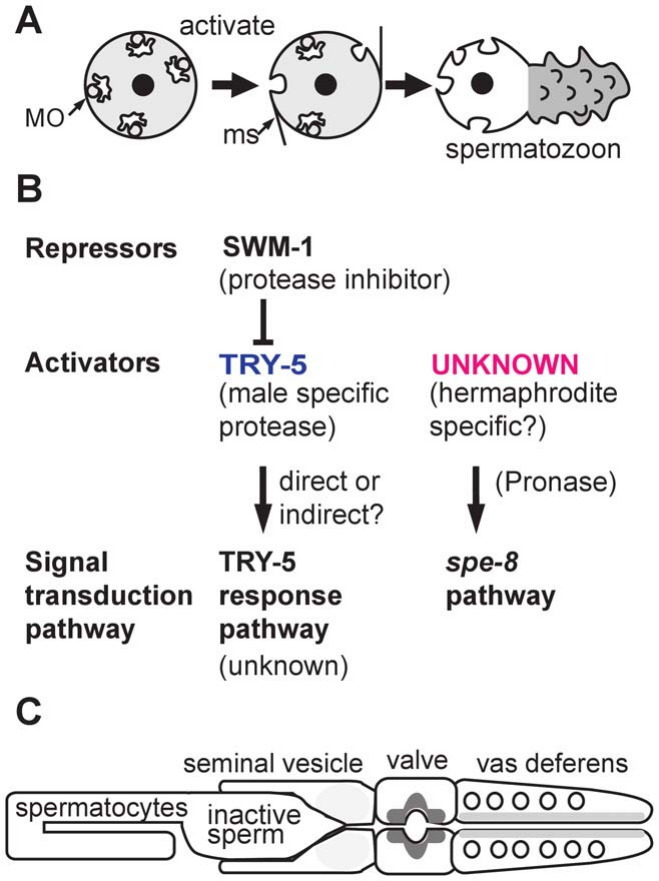
Sperm activation in *C. elegans*. (A) Sperm activation involves the initial formation of microspikes, the fusion of membraneous organelles (MOs) with the plasma membrane, and the extension of motile pseudopods. (B) In males, SWM-1 prevents precocious activation of stored sperm by inhibiting the male-specific, extracellular factor TRY-5. Active TRY-5 triggers sperm activation through an unknown signal transduction pathway. In hermaphrodites, sperm are activated via an unknown activator that acts through the *spe-8* signal transduction pathway. Both male and hermaphrodite sperm possess the two distinct signal transduction pathways (bottom row). The seminal fluid component TRY-5 can transactivate hermaphrodite sperm that have defects in the *spe-8* group genes. In one model, the activator of the *spe-8* signal transduction pathway is hermaphrodite-specific and activates *try-5* male sperm within the uterus. In an alternative model, the activator of the *spe-8* pathway is expressed in both hermaphrodites and, redundantly, in males. (C) Diagram of the *C. elegans* male gonad with the relative amount of TRY-5 within cells of the seminal vesicle, valve, and vas deferens indicated by different levels of gray shading.

In the game of evolutionary fitness, males must maximize the chance that their sperm successfully fertilize oocytes. Species-specific strategies include making more, bigger, or faster sperm, or producing seminal fluid that does more than serve as a vehicle for sperm transfer [1]. Importantly, seminal fluid components not only modulate sperm function and promote their competitiveness and long-term viability, but also initiate various physiological changes within the female such as increasing her rate of ovulation and decreasing her receptivity to other males [2]–[4]. Adding to this complexity, ejaculate is often generated in a series of compositionally distinct spurts resulting from the sequential emptying of various sexual glands [2], [5]. Yet how most seminal proteins function, particularly in the context of sperm activation (Figure 1A), remains unclear.

Even less is known about how reproductive fitness is maximized when both sexes (males and hermaphrodites) produce sperm. Certainly male and hermaphrodite *Caenorhabditis elegans* sperm face distinct challenges. In hermaphrodites, sperm are produced first, before their gonad switches over to exclusively producing oocytes [6]. Hermaphrodite sperm activate to form motile spermatozoa as they are physically pushed into the spermatheca by ovulating oocytes. Once there, they wait for fertilization opportunities as individual oocytes enter the spermatheca in an assembly-line fashion. Typically, every sperm gets an oocyte, and their only challenge is to remain in the spermatheca as the newly fertilized oocytes squeeze through the spermatheca on their way to the uterus [7].

In contrast, the larger male sperm are stored in a quiescent state within the seminal vesicle and only activate to form motile spermatozoa during the process of ejaculation. After insemination, male sperm must migrate from the vulva to the spermatheca and then out-compete the hermaphrodite's own sperm [8], [9].

Three lines of evidence suggest that *C. elegans* sperm activation is regulated in a sex-specific manner:

1. Molecular genetic studies identified components of a sperm-specific SPE-8 signal transduction cascade that function in both male and hermaphrodite sperm but are only essential for the activation of the hermaphrodite's own sperm (Figure 1B) [10]–[14]. Although mutant hermaphrodites are self-sterile, their sperm can be trans-activated by seminal fluid from either wild-type or *spe-8* males. Conversely, mutant males are fertile, but their sperm activates abnormally in response to in vitro activation by the protease Pronase. Together, these results suggest that although the sperm activators are expressed in a sex-specific manner, both male and hermaphrodite sperm retain the capacity to respond to either activator.
2. Conversely, the somatically expressed protease inhibitor SWM-1 is specifically required for male, but not hermaphrodite, fertility [15]. SWM-1 specifically regulates the timing of sperm activation in males; in the absence of SWM-1, male sperm activate precociously within the seminal vesicle, precluding their efficient transfer to hermaphrodites.
3. Lastly, sperm activation was found to be a key factor in the evolution of hermaphroditism, as females from the male/female species *Caenorhabditis remanei* were experimentally transformed into hermaphrodites simply by lowering the activity of two genes: SWM-1 and the “female” promoting factor TRA-2 [16].

How do these findings fit together? In this issue of *PLoS Genetics*, Smith and Stanfield [17] provide new insights regarding two partially redundant signal transduction pathways that regulate *C. elegans* sperm activation.

## TRY-5 Is a Sex-Specific Component of the Male Sperm Activation Pathway

Since male sperm activation can be triggered in vitro by proteases and blocked in vivo by the protease inhibitor SWM-1, the authors hypothesized that the male-specific sperm activator might be both a protease and a direct target of SWM-1. Taking advantage of both forward and reverse genetic approaches, they screened for mutations that suppress precocious sperm activation in *swm-1* males and identified a single, secreted trypsin-class serine protease (TRY-5), whose depletion suppressed the precocious activation and transfer defects of *swm-1* male sperm.

To further characterize this putative male sperm activator, the authors examined the effect of depleting TRY-5 alone, or in combination with previously characterized sperm activation mutants (Figure 1B). Since TRY-5 is expressed exclusively in males, it was not surprising that *try-5* hermaphrodites were fertile. However, the discovery that *try-5* males were also fertile suggested that their sperm were being activated via a second, TRY-5-independent pathway. Suspecting that this second pathway involved both the *spe-8* signal transduction pathway and trans-activation by the hermaphrodite-specific activator, the authors demonstrated that mutant males lacking both TRY-5 and *spe-8* class genes were infertile and that seminal fluid from *try-5* males could not transactivate *spe-8* hermaphrodite sperm. The authors concluded that, in *C. elegans*, sperm activation is controlled by two partially redundant pathways: an unknown signal transduction pathway triggered by the male-specific TRY-5 and the *spe-8* signal transduction pathway triggered by an unknown hermaphrodite-specific activator.

## TRY-5 Is a Component of Seminal Fluid

If TRY-5 functions directly as the sperm activator, it should be present in the seminal fluid, specifically during the process of ejaculation. To test whether TRY-5 is expressed at the correct time and in the correct place, the authors generated transgenic lines containing either a Ptry-5::GFP reporter or a Ptry-5::TRY-5::GFP fusion construct. Sure enough, *try-5* was expressed specifically within the cells of the somatic male gonad that surround the exit path of the sperm (Figure 1C). Furthermore, prior to ejaculation, TRY-5 localized to secretory vesicles within these somatic cells. During ejaculation, TRY-5 was secreted as a component of the seminal fluid and was ejaculated in a reproducible pattern of spurts from distinct regions of the somatic gonad. The dynamics of TRY-5 secretion and transfer suggest that male sperm activation may occur within the uterus of the hermaphrodite rather than within the vas deferens of the male.

## Answers and More Questions

The discovery and characterization of the protease TRY-5 as the putative male-specific sperm activator addresses both how and why male and hermaphrodite sperm are activated in a sex-specific manner. For male sperm, activation must be both robust and tightly coordinated with each ejaculation event. For hermaphrodite sperm, activation may be a one-time event and robustness may not be as critical. As TRY-5 is the first sex-specific component of the sperm-activation pathway to be identified, it will be critical both for elucidating the elements of the downstream signal transduction machinery and for addressing broader questions regarding the independent evolution of hermaphroditism.

At the same time, this study raises several new questions. Does TRY-5 activate sperm directly or does it function as part of a protease cascade? What are the sperm-expressed targets of the TRY-5 activation pathway? What is the hermaphrodite-expressed activator that functions upstream of the SPE-8 signal transduction pathway? Finally, are there really distinct male and hermaphrodite sperm activators, or do males normally express multiple activators while hermaphrodites express only the one that triggers the SPE-8 pathway (Figure 1B)?

Additionally, this is the first study to reveal *C. elegans* as a model for elucidating the temporal and physiological dynamics of seminal fluid production, as the TRY-5::GFP fusion protein can be directly observed through the transparent body of the worm. Future studies could use similar approaches to investigate the secretory dynamics of other seminal fluid proteins.
